# Charting a landmark-driven path forward for population genetics and ancient DNA research in Africa

**DOI:** 10.1016/j.ajhg.2024.05.019

**Published:** 2024-07-11

**Authors:** Elizabeth A. Sawchuk, Kendra A. Sirak, Fredrick K. Manthi, Emmanuel K. Ndiema, Christine A. Ogola, Mary E. Prendergast, David Reich, Eva Aluvaala, George Ayodo, Lamine Badji, Nancy Bird, Wendy Black, Rosa Fregel, Njeri Gachihi, Victoria E. Gibbon, Agness Gidna, Steven T. Goldstein, Reem Hamad, Hisham Y. Hassan, Vanessa M. Hayes, Garrett Hellenthal, Solomon Kebede, Abdikadir Kurewa, Chapurukha Kusimba, Elizabeth Kyazike, Paul J. Lane, Scott MacEachern, Diyendo Massilani, Emma Mbua, Alan G. Morris, Christina Mutinda, Freda Nkirote M’Mbogori, Austin W. Reynolds, Sarah Tishkoff, Miguel Vilar, Getnet Yimer

**Affiliations:** 1 Cleveland Museum of Natural History, Cleveland, OH, USA; 2 Department of Anthropology, University of Alberta, Edmonton, AB, Canada; 3 Department of Anthropology, Stony Brook University, Stony Brook, NY, USA; 4 Department of Human Evolutionary Biology, Harvard University, Cambridge, MA, USA; 5 Department of Genetics, Harvard Medical School, Boston, MA, USA; 6 National Museums of Kenya, Nairobi, Kenya; 7 Department of Anthropology, Rice University, Houston, TX, USA; 8 Broad Institute of Harvard and MIT, Cambridge, MA, USA; 9 Howard Hughes Medical Institute, Harvard Medical School, Boston, MA, USA; 10 Kenya Medical Research Institute, Nairobi, Kenya; 11 Jaramogi Oginga Odinga University of Science and Technology, Bondo, Kenya; 12 Cultural Engineering Research Unit (URICA) of IFAN-University Cheikh Anta Diop, Dakar, Senegal; 13 UCL Genetics Institute and Research Department of Genetics, Evolution, and Environment, University College London, London, UK; 14 Archaeology Unit, Department of Research & Exhibitions, Iziko Museums of South Africa, Cape Town, South Africa; 15 Human Evolution Research Institute, University of Cape Town, Cape Town, South Africa; 16 Evolution, Paleogenomics and Population Genetics Group, Department of Biochemistry, Microbiology, Cell Biology and Genetics, Universidad de La Laguna, San Cristóbal de La Laguna, Santa Cruz de Tenerife, Spain; 17 Division of Clinical Anatomy and Biological Anthropology, Department of Human Biology, University of Cape Town, Cape Town, South Africa; 18 Department of Cultural Heritage, Ngorongoro Conservation Area Authority, Arusha, Tanzania; 19 Department of Anthropology, University of Pittsburgh, Pittsburgh, PA, USA; 20 Diversity and Diseases Group, Institute of Endemic Diseases, University of Khartoum, Khartoum, Sudan; 21 Bahrain Defence Force Hospital, Royal Medical Services, Riffa, Kingdom of Bahrain; 22 School of Medical Sciences, University of Sydney, Sydney, NSW, Australia; 23 School of Health Systems and Public Health, University of Pretoria, Pretoria, South Africa; 24 Authority for Research and Conservation of Cultural Heritage Ethiopia, Addis Ababa, Ethiopia; 25 Department of Anthropology, University of Florida, Gainesville, FL, USA; 26 Department of Anthropology, University of South Florida, Tampa, FL, USA; 27 Department of History, Archaeology and Heritage Studies, Faculty of Arts and Humanities, Kyambogo University, Kampala, Uganda; 28 Department of Archaeology, University of Cambridge, Cambridge, UK; 29 School of Geography, Archaeology and Environmental Studies, University of the Witwatersrand, Johannesburg, South Africa; 30 Department of Archaeology and Anthropology, Duke Kunshan University, Kunshan, China; 31 Department of Genetics, Yale School of Medicine, Yale University, New Haven, CT, USA; 32 Department of Human Biology, University of Cape Town, Cape Town, South Africa; 33 Department of Microbiology, Immunology, and Genetics, School of Biomedical Sciences, University of North Texas Health Science Center, Fort Worth, TX, USA; 34 Department of Genetics, University of Pennsylvania, Philadelphia, PA, USA; 35 Penn Center for Global Genomics & Health Equity, Perelman School of Medicine, University of Pennsylvania, Philadelphia, PA, USA; 36 Department of Biology, University of Pennsylvania, Philadelphia, PA, USA; 37 Department of Anthropology, University of Maryland, College Park, MD, USA

**Keywords:** Africa, genetics research, population history, capacity building, community engagement

## Abstract

Population history-focused DNA and ancient DNA (aDNA) research in Africa has dramatically increased in the past decade, enabling increasingly fine-scale investigations into the continent’s past. However, while international interest in human genomics research in Africa grows, major structural barriers limit the ability of African scholars to lead and engage in such research and impede local communities from partnering with researchers and benefitting from research outcomes. Because conversations about research on African people and their past are often held outside Africa and exclude African voices, an important step for African DNA and aDNA research is moving these conversations to the continent. In May 2023 we held the DNAirobi workshop in Nairobi, Kenya and here we synthesize what emerged most prominently in our discussions. We propose an ideal vision for population history-focused DNA and aDNA research in Africa in ten years’ time and acknowledge that to realize this future, we need to chart a path connecting a series of “landmarks” that represent points of consensus in our discussions. These include effective communication across multiple audiences, reframed relationships and capacity building, and action toward structural changes that support science and beyond. We concluded there is no single path to creating an equitable and self-sustaining research ecosystem, but rather many possible routes linking these landmarks. Here we share our diverse perspectives as geneticists, anthropologists, archaeologists, museum curators, and educators to articulate challenges and opportunities for African DNA and aDNA research and share an initial map toward a more inclusive and equitable future.

## Teaser

Moving toward an increasingly inclusive, equitable, and engaged future for population history-focused African DNA and aDNA research requires identifying existing barriers and proposing solutions to overcome them. We create a vision for the next decade of research and identify the landmarks that will collectively bring us closer to this goal.

## Introduction

The African continent is the most genetically diverse on the planet when it comes to our species,^1^ and there has been surging interest in human DNA and ancient DNA (aDNA) research, especially within the last few years. The analysis of DNA from present-day people has been transformed by a deeper appreciation of the power of increasingly dense sampling of genetic information. This has facilitated a more nuanced understanding of the diversity and fine-scale structure that exists within geographically constrained areas, enabling more detailed reconstructions of demographic histories.^2^^,^^3^^,^^4^^,^^5^^,^^6^^,^^7^ These densely collected data are improving imputation, fine-mapping, and polygenic risk score analysis for African people.^8^^,^^9^ African aDNA research has similarly grown rapidly in the last decade, despite early pessimism regarding elevated rates of biomolecular degradation in hot and humid environments. Following the publication of the first genome-wide data from an ancient African in 2015,^10^ genomic data from hundreds of ancient African individuals have challenged some existing narratives of population histories and sparked new questions for geneticists, archaeologists, linguists, and other scholars on both regional and continental scales.^11^^,^^12^^,^^13^ While African sequences still only account for ∼3% of ancient genomes published to date, the exponential rate of growth is similar to that of other world regions.^14^ However, this growth has not occurred evenly across the continent, and some countries or regions still lack any aDNA data.

Given the trajectory of genetics research, now is an ideal time to survey the current research landscape in Africa and consider future challenges. Despite growing international interest in human genomics research on the continent, African researchers are starkly underrepresented. Part of the problem is that many African scholars face barriers to accessing and integrating into the spaces where genetics projects are planned and carried out.^15^ Limited laboratory infrastructure on the continent combined with financial disparities and visa requirements that complicate travel to professional meetings usually held in the Global North reduce leadership opportunities in genomics research for African scholars. Members of local communities, museum curators, heritage officials, and others who have a vested interest in research are rarely central to research projects, despite their critical labor in providing samples and/or curating them. Furthermore, museological practices in most African countries are entangled with colonial legacies that discourage the participation of local communities in the collection, curation, and management of their heritage resources, including genetic resources.^16^^,^^17^^,^^18^ Although there have been efforts toward the establishment and growth of DNA and aDNA research in Africa, most African institutions still lack the capacity and infrastructure for genetic studies to become deeply entrenched. The prohibitively high cost of establishing and maintaining laboratory spaces and equipment limits the amount of work that can be carried out on the continent and creates power imbalances between those based at well-funded laboratories in the Global North and African researchers interested in engaging with genetic work. Additional challenges include a lack of access to journals where research is published and a history of exploitative research—in many fields—by non-African scholars.^19^^,^^20^ The cumulative result is that conversations about African peoples and their past are usually held outside Africa and to the exclusion of African scholars and voices. An important step forward in achieving an ethical and equitable future for African DNA and aDNA research is moving these conversations to Africa and involving those who have been historically sidelined.

### The DNAirobi workshop

In May 2023, we organized a human population history-focused DNA and aDNA workshop in Nairobi, Kenya. Hosted by the National Museums of Kenya, “DNAirobi” was born from the recognition that carrying out future DNA and aDNA research in Africa requires holding discussions on African soil where African colleagues from across the continent and with different specialties, including genetics, archaeology, anthropology, education, and museum curation, can contribute their perspectives. For three days, scholars delivered presentations and participated in roundtable discussions that brought to light challenges inherent in this work and inspired ideas for a better future. In total, the workshop was attended by ∼100 people, 80% of whom were based in Africa.

In this paper, we share key points garnered from our conversations. While ethics and related matters such as equity, inclusiveness, and capacity building were central to our discussions, what emerged was not a distinct set of guidelines for DNA and aDNA research in Africa. For that, we look to recent best practice papers, many of which have substantial overlap: for example, that all genetics research should be community engaged, sustainable, inclusive of a diverse range of collaborators, and accessible to all.^20^^,^^21^^,^^22^^,^^23^^,^^24^^,^^25^^,^^26^^,^^27^^,^^28^^,^^29^^,^^30^ However, these papers often lack guidance as to the best way to put these principles into practice, and there has been little discussion of how these broad concepts should be applied in different regions of the world. Existing recommendations written specifically for African contexts (e.g., Gibbon^20^ and Prendergast and Sawchuk^21^) represent a limited range of perspectives and would benefit from broader inclusion of African scholars. Therefore, what emerged most prominently in our discussions was the need to share ideas generated by a diverse group of people—including many whose voices have been historically underrepresented—on how to move toward an increasingly inclusive, equitable, and engaged future for population history-focused African DNA and aDNA research by identifying existing barriers and proposing solutions to overcome them.

We do not expect a singular path forward for DNA and aDNA research on a continent as complex and diverse as Africa, just as we do not believe any path will or should remain static over time.^31^ Instead, as the research landscape evolves alongside science and society, we must iteratively and collectively determine how to adapt. To do this, we propose a map that follows landmarks through a shifting landscape. We define “landmarks” as points of consensus that emerged throughout our discussions in response to the challenges encountered on the way to achieving an inclusive and equitable future for genomic research. The course between these landmarks can and will change depending on context and through time, but all need to be visited as part of the journey. This presents opportunities to traverse the landscape in new ways. That said, creating a map is a daunting task, as the paucity of genomics research in Africa relative to other parts of the world, combined with a history of extractive research, limits experiences and policies to draw upon. We acknowledge that DNAirobi was only an initial meeting and there is a need for resources to enable such meetings to occur regularly and in other parts of Africa, in concert with other efforts toward networking, capacity building, and knowledge sharing.

As we begin to identify the landmarks on our map, we pose the following key questions. (1) What should DNA and aDNA research in Africa look like in ten years? (2) What are the gaps between this ideal and our current reality? And (3) where should we focus our efforts to reach this future?

### A vision for population history-focused DNA and aDNA research in Africa in ten years’ time

A landmark-driven map must have a destination and a timeline. Within the next ten years, the ideal “destination” we seek is a place where the following are true:

(1) Leadership. African scholars are leading and driving DNA and aDNA research. This is reflected through lead and senior authorship on publications and principal investigator status in labs and on grants. Expert knowledge, diverse skills, and technology required to lead research are fluidly transferred among researchers living across continents and around the world.
(2) Heritage conservation. Conservation of heritage is prioritized and DNA and aDNA research contributes to the creation of necessary infrastructure to preserve and celebrate heritage. Institutions and groups that safeguard and manage heritage are sustainable in the long-term, with resources to build the infrastructure and the ability to set and enforce policies and regulations.
(3) Partnerships. Collaborations reflect equal partnerships among scholars, including those that involve partners from the Global North. All partners feel equitable investment in the project and ownership of research products.
(4) Training and capacity building. Funding mechanisms exist for African researchers to pursue top-level educational opportunities and experiences. Trainees have opportunities locally, across Africa, and abroad, and those who wish to pursue advanced degrees are supported by an international network of scholars and institutions. The initiation of training programs in Africa makes it easier and more affordable to access education adapted for local contexts and begins to improve discipline-specific capacity building across Africa.
(5) Community engagement and support. Community members who may draw meaning from research results (which can include but are not limited to descendant, guardian, and other “stakeholder” communities) are met at the times and in the spaces they choose, are engaged in ways that are meaningful to them, and are supported in deriving knowledge from scientific data rather than just giving consent. Researchers respect the values, norms, and religious and cultural practices of communities. DNA and aDNA researchers recognize challenges faced by partner communities and respect their knowledge, time, and resources while realistically communicating potential benefits which may or may not align with community priorities.
(6) Effective communication. DNA and aDNA research is effectively shared with a wide range of people, including specific communities who may draw personal connections to results, as well as school-aged children, policy makers, and the general public. Questions of whom to engage and how to make information accessible and understandable to them are addressed during the initial stages of research project development and periodically re-evaluated. Results are shared in ways that are cognizant of privacy concerns while being supportive of science communication and literacy and creating possibilities for growth in associated disciplines.

### Synthesizing gaps between the present situation and the future to which we aspire

In sketching out a vision for the future of DNA and aDNA research in Africa, the gaps between where we are and where we want to be are evident. In addition to recognizing challenges faced by individual African researchers and institutions to accessing knowledge and infrastructure, we must also consider the realities of unstable political and economic situations in many African countries directly affecting scientific research and acknowledge that research may not be a priority for some governments and communities. Current challenges cut across multiple arenas, from geopolitical circumstances and scientific climate to mechanisms for public communication and community support. Efforts to overcome these challenges to date have been carried out by diverse individual African and non-African researchers, labs, and institutions under the umbrella of “capacity building”; however, reaching our destination requires structural changes that go beyond grassroot efforts.

Discussions at the workshop coalesced around the creation of a dynamic research ecosystem in which all partners contribute to research projects in the ways they choose and all contributions are equally valued. This requires effectively reducing or eliminating power and resource imbalances so that researchers studying the African past and present can collaborate with one another and access whatever tools they may need to achieve their aims. In this ecosystem, power is decentralized and comes from a range of sources. The resulting research is not only diverse, engaged, and multi-scalar, but is also sustainable and dynamic in the long term in response to changes in science and society.

Creating such a research ecosystem was seen by participants as more desirable in the short and immediate term than, for example, establishing a population history-focused DNA or ancient DNA lab on the continent that was responsible for carrying out all steps of data generation and analysis. This was seen as unrealistic in general for a number of reasons, but especially in light of presently available resources for staff and maintenance costs. Furthermore, what appear to be immediate infrastructure needs now may be obsolete in a few years’ time as new models for data processing and generation become available and accessible. However, because developing laboratories and computational infrastructure was still seen as important for the ultimate goal of achieving scientific independence, steps may be taken en route toward a longer-term goal. For example, the identification, selection, and processing of samples could be led by African scholars and take place in local laboratories and regional research units as they build capacity to also lead analysis and interpretation of data. There are multiple pathways for work to happen in ways that still allow for research leadership and capacity building and are sustainable in the long term.

The interest in DNA and aDNA research across Africa is clear, and the demand for increased equity and partnership grows. How then do we create an equitable research ecosystem in which scholars and engaged communities organically come together to create knowledge, while avoiding situations that perpetuate existing power imbalances and exclusionary practices? We propose the following areas on which to concentrate.

### Landmarks on the way to our desired future

#### Landmark 1. Improving communication: The interest is there, but the messaging is off

There is a stark difference between communication that counts as academic “currency” (i.e., peer-reviewed publications) and communication that effectively reaches people who may draw meaning from the results of a scientific study. The academy is currently structured so that the former is more highly valued, and accordingly scientific publications have become the fundamental currency that permits research to continue and expand, allowing researchers to obtain promotions and secure new funding and consequently shaping communication strategies for genetics work. A further consideration is the transient and precarious nature of early career research positions wherein scholars not only have fewer resources, but also a more limited time frame in which to establish relationships and return results outside of academic publications. Nevertheless, a recurring point at the workshop was that the scientific community needs to prioritize communication with diverse audiences, in the same way that we value communications which fill our academic bank. A focus should be on overall scientific literacy in concert with efforts to reach people in positions of power (such as policy makers, government officials, and peer scientists and professionals), as well as the broader public, including communities relevant to research. Communication should be conducted in ways that allow people to comprehend, embrace, and draw meaning from research. Without holistic and effective communication strategies developed in close partnership with local collaborators, it is difficult for people to become invested in any kind of research.

What should this communication look like? One strategy for returning results, which we applaud, is the preparation of a handout of key conclusions written without scientific jargon and translated into appropriate language(s), both those of descendant communities/community partners and, if necessary, national languages. But we also need to go beyond this, and research partners must work together—in a way that learns from engagement with descendant groups and/or community partners who are local to the area under study—to explore alternative means for communication. For example, for many of the groups we study, orality has more scope than written channels. Other effective strategies can include in-person or digital art, museum exhibits, dramatic productions, and songs or dances that communicate findings.^32^^,^^33^ Results dissemination may be tied with important events and celebrations that have practical meaning to the community, such as market days, festivals, and fairs. Additionally, researchers may look to developing curricula that allows people of all ages and educational backgrounds to better engage with and relate to the message being conveyed, and/or popularize findings by giving press conferences and involving local media outlets. Existing guidelines developed in Africa for community engagement with genomic research, including those by Human Heredity & Health in Africa (H3Africa^34^), may provide a useful template.^35^^,^^36^

A further point raised at the workshop is that researchers must carefully consider their phrasing and approaches when engaging communities about research and be inclusive of people who may not fit narrow definitions of descendants. Our discussions raised concerns about the use of terms like “ancestor” to describe human remains and “indigenous” as a marker of identity^25^ with many African and other scholars wary of the implications of such language despite caveats about not implying genealogical relationships.^37^ Complex legacies of colonial and post-colonial displacements and violence as well as oral histories of recent migration mean that many contemporary groups do not identify as the descendants of geographically proximate archaeological and historical populations.^32^^,^^38^^,^^39^ It is harmful to exclude people or engage them using language that they find alienating. Similarly, it is harmful to assume that such groups would be disinterested in this research. While in some places there is a deep distrust of genetic research due to the legacy of race-based scientific investigation and extractive practices, in other places there is a strong desire for greater involvement in DNA and aDNA research. Researchers should not attempt to provide a re-education based on genetic data; instead, they should accurately communicate research findings while also being attentive to basic scientific literacy needs and paying respect to existing systems of knowledge, which may or may not be scientifically based, as well as the structures under which that knowledge is disseminated. All of this requires understanding and appreciating what the community knows about themselves and how they wish to be addressed and engaged. Considerations of the space, timing, and intentions of communication are as important as the content, necessitating ongoing discussions with communities and community leaders regarding the structure of engagement and reporting starting from the earliest stages of research.^40^

Effective communication is multi-modal and requires a network of people who are dedicated to incorporating new results into existing knowledge systems and carrying out ongoing evaluation (e.g., through community surveys) to assess whether efforts are producing the desired results.^41^ In time, these efforts may become encoded in policy and legislation that re-center and protect communal interests, traditional knowledge, and cultural expressions that are directly or indirectly related to genetic resources. Finally, it is important to reduce barriers to communication and collaboration with African scholars, including publishing open access and holding lab meetings and workshops virtually when they cannot be held in Africa.

#### Landmark 2. Reframing equitable relationships: Bring what you have, take what you need

While there have been examples of strong partnerships between Global North and Global South scholars, scientific research has also included significant “parachute” or “helicopter” research reflecting differential contributions of resources, personnel, and skill sets that led to imbalanced relationships.^19^^,^^42^^,^^43^^,^^44^^,^^45^ Although such extractive practices are now widely critiqued, research still operates within a system where individuals and/or institutions from the Global North provide the vast majority of financial support for projects and take the lead on developing, executing, and disseminating the results, while African individuals and institutions often facilitate sampling and make connections on the ground. Rectifying this imbalance will require a drastic and sustained increase in funding and capacity building for African researchers, including fully supported training opportunities, to position them in lead roles in international scientific projects. Although reaping the benefits of expanded capacity building initiatives will take time, there is immediate need to reframe relationships, including reevaluating the resources brought to these relationships.

Reframing relationships and creating equitable partnerships seems relatively straightforward at a superficial level. For example, we can speak of all parties in a research project as partners who are equally essential to the project’s success. However, reframing relationships at a deeper level requires appreciating that desired outcomes and benefits are different in different spheres. Conversations at the workshop made it evident that celebrating ancestry, raising awareness for conservation/preservation of cultural heritage, and providing appropriate benefits for contributions to knowledge production (which may include sharing of material benefits) may be priorities for people outside of the academic ecosystem. The primary and immediate needs of communities should be considered before other benefits. No one partner should project their own desires onto the other partners; instead, all should be met where they are and have the opportunity to express what they want and need from the partnership. To this end, the call for more capacity building and training opportunities for African scholars cannot be overstated.

Partnerships are rarely 50/50 at all times. Instead, partners bring what they have to the table, and take what they need from it. Our discussions called for such equitability to guide our research relationships as well. Persistent and profound inequalities in funding structures and currency exchange rates make it likely that, in the immediate future, individuals and institutions from the Global North will continue to control the vast majority of financial resources and the venues for DNA and aDNA data to be produced. Africa must be recognized and appreciated as home to the invaluable cultural and biological heritage that is the starting point of this work. In addition to calling for more funding for African scholars, we must embrace the differences and fluctuations in resources, personnel, capacity, and interest, recognizing that research cannot proceed without funding nor without biological/cultural heritage. DNA and aDNA projects already provide a model for this kind of relationship, where collaborators with diverse backgrounds and skill sets variably contribute to planning, sampling, and data generation, analysis, and interpretation throughout the life of a study.^46^ Contributing different strengths and resources produces something greater than all individual parts and increases the impact of research.

#### Landmark 3. Science for society: What else can research create?

A final theme was the importance of holding internationally attended meetings, conferences, and workshops in Africa and the overall need for greater investment in Africa’s scientific infrastructure. It is difficult for most African scholars, especially students, to attend events in the Global North and even across the African continent because of financial and visa limitations. However, continuing to discuss African research outside Africa, or only considering certain parts of the continent, does not promote an equitable future. While there are important Africa-based professional associations and meetings (e.g., H3Africa and the African Society for Human Genetics [AfSHG] for genomics, and the PanAfrican Archaeological Association), these have not historically taken on ancient population history research as derived from genomics.

Our discussions sparked interest in the broader kinds of benefits investment in African research will bring. At the individual and community levels, increased scientific literacy and educational and employment opportunities will offer manifold opportunities for economic and social advancement. As in the Global North, fostering an interest in STEM creates a pathway to scientific careers and improves the economic outlook for entire communities.^47^^,^^48^ Studies of modern DNA with community consent can offer additional tangible benefits, such as better understanding of genetic predisposition to disease in families and communities, with the possibility of future applications to personalized medicine, which is still in its infancy around the world.^49^^,^^50^^,^^51^ Finally, increased interest in specific communities and regions may bring further benefits, such as improvements to local infrastructure and opportunities for student mentorship, curriculum development, and heritage engagement. Importantly, such outcomes would support continent-wide strategic planning such as the African Union Agenda 2063.^52^

There was enormous interest in the possibility of establishing a network or consortium of African institutions involved in both DNA and aDNA research that could function independently of Global North collaborators. DNAirobi provided a unique setting for broader discussions on common issues that universities, labs, and museums were facing related to finding funding, facilitating capacity building, and curating archaeological and fossil collections. It is critical for African institutions to be able to function independently and set and enforce their own research and heritage agendas in order to continue the work of disengaging from colonial origins and legacies. Creating a more equitable ecosystem in one area of science and research will inevitably have cascading effects by empowering African scholars to work collaboratively to identify shared problems, distribute resources, and innovate solutions that are grounded in and meet the specific needs of the African research landscape. Such networks offer the best chances for long-term institutional resilience in the face of coming changes.

Finally, we emphasize the potential global benefits of achieving our goals. Increased equity, diversity, and inclusion in research will improve our work by increasing the number and variety of voices contributing to interpretation of complex phenomena.^53^ Africa is the most genetically and ethnolinguistically diverse continent on the planet; these same qualities that attract genomic researchers to Africa illustrate the need for an increasingly diversified workforce. Africa’s genetic diversity, past or present, remains poorly studied, with entire regions, time periods, and lines of questioning left out of current scholarship. Historically underfunded African museums and universities safeguard the most valuable resources we have for understanding human origins and population history, and play a key role in combating the disappearance of heritage sites.^18^^,^^54^ When it comes to aDNA, there will always be challenges with recovery in hot and humid climates, but it is prudent to remember how much has changed in the past decade and support the African institutions and scholars who hold irreplaceable resources in trust for all of us.

### Conclusions

Africans are the primary knowledge holders of African samples, data, and historical contexts. A takeaway from our workshop is that many African scholars want more access to DNA and aDNA research within a context of equitable partnerships, acknowledgment, and representation, and capacity building that ultimately contributes to more African-led research in the future. Creating this ecosystem, in which ethical and equitable research thrives and adapts to inevitable changes in our world, necessitates structural changes to the very way that science is designed and supported.

The body of best practice literature for DNA and aDNA research frequently focuses on the importance of community engagement, but we rarely discuss how professional and community networks are created and fostered. Ideally, members of relevant communities are aware of and empowered to engage with scientific research and take on leader or partner roles. More frequently, non-geneticist collaborators like anthropologists, archaeologists, physicians, government representatives, and museum curators identify relevant contacts and communities and act as “boots on the ground” to facilitate engagement.^21^ While this emphasizes the importance of building inter- and multidisciplinary teams,^55^^,^^56^ such partnerships are still restricted to people within existing networks or who are otherwise accessible to researchers, many of whom are still from the Global North. There is inherent tension between genomic research as a relatively fast-moving science where lab funding dictates the timeline and need to publish results, and the work of meaningfully engaging communities which is a slow, often decades-long process that relies on building trust and establishing effective channels for ongoing communication. In the absence of infrastructure that supports effective community engagement, research can quickly become technocratic, alienated, and alienating.^57^

The bottom line is that until we have infrastructural support to create the desired equitable ecosystem, we cannot expect to effectively implement best practices for African DNA and aDNA research. We encourage larger labs that command more funding to continue and expand their efforts toward capacity building and equitable co-design of research with Africa-based partners. However, we also need funding structures to dedicate more resources to African scholars either directly or in cooperation with institutions that are committed to providing training and capacity building opportunities, recognizing that such an investment has the potential to set off a ripple effect of positive growth on a multitude of scales. African scholars can more effectively mediate relationships with their own communities (already coming from a position of trust), leading to stronger long-term relationships that go beyond fleeting contact to obtain consent and report results. Effective community engagement requires healing deep-rooted structural inequalities, starting with providing better access to basic education for all, including girls and other marginalized groups, and improving scientific literacy beginning with the youngest students so they grow up knowing what is possible. Given the enormous diversity of people and potential research questions across the African continent, the result will be an ecosystem of empowered people who create and derive benefits from scientific knowledge.

Building truly equitable partnerships requires investing in entire societies and next generation scholars, work that will take decades and have impacts that extend well beyond genetic research. The first step is appreciating the scale of this effort, and identifying the landmarks along the way that will bring us closer to this goal.

## References

[1] Tishkoff S.A., Reed F.A., Friedlaender F.R., Ehret C., Ranciaro A., Froment A., Hirbo J.B., Awomoyi A.A., Bodo J.M., Doumbo O. (2009). The Genetic Structure and History of Africans and African Americans. Science.

[2] Fan S., Spence J.P., Feng Y., Hansen M.E.B., Terhorst J., Beltrame M.H., Ranciaro A., Hirbo J., Beggs W., Thomas N. (2023). Whole-genome sequencing reveals a complex African population demographic history and signatures of local adaptation. Cell.

[3] Sherman R.M., Forman J., Antonescu V., Puiu D., Daya M., Rafaels N., Boorgula M.P., Chavan S., Vergara C., Ortega V.E. (2019). Assembly of a pan-genome from deep sequencing of 910 humans of African descent. Nat. Genet..

[4] Choudhury A., Aron S., Botigué L.R., Sengupta D., Botha G., Bensellak T., Wells G., Kumuthini J., Shriner D., Fakim Y.J. (2020). High-depth African genomes inform human migration and health. Nature.

[5] Fatumo S., Yakubu A., Oyedele O., Popoola J., Attipoe D.A., Eze-Echesi G., Modibbo F.Z., Ado-Wanka N., 54gene Team, NCD-GHS Consortium (2022). Promoting the genomic revolution in Africa through the Nigerian 100K Genome Project. Nat. Genet..

[6] Bird N., Ormond L., Awah P., Caldwell E.F., Connell B., Elamin M., Fadlelmola F.M., Matthew Fomine F.L., López S., MacEachern S. (2023). Dense sampling of ethnic groups within African countries reveals fine-scale genetic structure and extensive historical admixture. Sci. Adv..

[7] López S., Tarekegn A., Band G., Van Dorp L., Bird N., Morris S., Oljira T., Mekonnen E., Bekele E., Blench R. (2021). Evidence of the interplay of genetics and culture in Ethiopia. Nat. Commun..

[8] Kamiza A.B., Toure S.M., Vujkovic M., Machipisa T., Soremekun O.S., Kintu C., Corpas M., Pirie F., Young E., Gill D. (2022). Transferability of genetic risk scores in African populations. Nat. Med..

[9] Martin A.R., Kanai M., Kamatani Y., Okada Y., Neale B.M., Daly M.J. (2019). Clinical use of current polygenic risk scores may exacerbate health disparities. Nat. Genet..

[10] Gallego Llorente M., Jones E.R., Eriksson A., Siska V., Arthur K.W., Arthur J.W., Curtis M.C., Stock J.T., Coltorti M., Pieruccini P. (2015). Ancient Ethiopian genome reveals extensive Eurasian admixture throughout the African continent. Science.

[11] Prendergast M.E., Sawchuk E.A., Sirak K.A. (2022). Genetics and the African Past. Oxford Research Encyclopedia of African History.

[12] Sirak K.A., Sawchuk E.A., Prendergast M.E. (2022). Ancient Human DNA and African Population History. Oxford Research Encyclopedia of Anthropology.

[13] Vicente M., Schlebusch C.M. (2020). African population history: an ancient DNA perspective. Curr. Opin. Genet. Dev..

[14] Mallick S., Micco A., Mah M., Ringbauer H., Lazaridis I., Olalde I., Patterson N., Reich D. (2024). The Allen Ancient DNA Resource (AADR) a curated compendium of ancient human genomes. Sci. Data.

[15] Prendergast M.E. (2023). Genetics and Archaeology. Afr. Archaeol. Rev..

[16] Ndlovu N., Smith B. (2019). The Past Is a Divided Country: Transforming Archaeology in South Africa. Afr. Archaeol. Rev..

[17] Karega-Munene (2011). Museums in Kenya: Spaces for Selecting, Ordering and Erasing Memories of Identity and Nationhood. Afr. Stud..

[18] Kusimba C., Klehm C., Mitchell P., Lane P. (2013). The Oxford Handbook of African Archaeology.

[19] Morris A.G. (2017). Ancient DNA comes of age, but still has some teenage problems. South Afr. J. Sci..

[20] Gibbon V.E. (2020). African ancient DNA research requires robust ethics and permission protocols. Nat. Rev. Genet..

[21] Prendergast M.E., Sawchuk E. (2018). Boots on the ground in Africa’s ancient DNA ‘revolution’: archaeological perspectives on ethics and best practices. Antiquity.

[22] Austin R.M., Sholts S.B., Williams L., Kistler L., Hofman C.A. (2019). Opinion: To curate the molecular past, museums need a carefully considered set of best practices. Proc. Natl. Acad. Sci. USA.

[23] Bardill J., Bader A.C., Garrison N.A., Bolnick D.A., Raff J.A., Walker A., Malhi R.S., Summer internship for INdigenous peoples in Genomics SING Consortium (2018). Advancing the ethics of paleogenomics. Science.

[24] Claw K.G., Anderson M.Z., Begay R.L., Tsosie K.S., Fox K., Garrison N.A., Summer internship for INdigenous peoples in Genomics SING Consortium (2018). A framework for enhancing ethical genomic research with Indigenous communities. Nat. Commun..

[25] Wagner J.K., Colwell C., Claw K.G., Stone A.C., Bolnick D.A., Hawks J., Brothers K.B., Garrison N.A. (2020). Fostering Responsible Research on Ancient DNA. Am. J. Hum. Genet..

[26] Fleskes R.E., Bader A.C., Tsosie K.S., Wagner J.K., Claw K.G., Garrison N.A. (2022). Ethical Guidance in Human Paleogenomics: New and Ongoing Perspectives. Annu. Rev. Genom. Hum. Genet..

[27] Sirak K.A., Sedig J.W. (2019). Balancing analytical goals and anthropological stewardship in the midst of the paleogenomics revolution. World Archaeol..

[28] Ali J., Cohn B., Mwaka E., Bollinger J.M., Kwagala B., Barugahare J., Sewankambo N.K., Ochieng J. (2021). A scoping review of genetics and genomics research ethics policies and guidelines for Africa. BMC Med. Ethics.

[29] De Vries J., Bull S.J., Doumbo O., Ibrahim M., Mercereau-Puijalon O., Kwiatkowski D., Parker M. (2011). Ethical issues in human genomics research in developing countries. BMC Med. Ethics.

[30] Rutakumwa R., De Vries J., Parker M., Tindana P., Mweemba O., Seeley J. (2019). What constitutes good ethical practice in genomic research in Africa? Perspectives of participants in a genomic research study in Uganda. Global Bioeth..

[31] Kaestle F.A., Horsburgh K.A. (2002). Ancient DNA in anthropology: Methods, applications, and ethics. Am. J. Phys. Anthropol..

[32] Alpaslan-Roodenberg S., Anthony D., Babiker H., Bánffy E., Booth T., Capone P., Deshpande-Mukherjee A., Eisenmann S., Fehren-Schmitz L., Frachetti M. (2021). Ethics of DNA Research on Human Remains: Five Globally Applicable Guidelines. Nature.

[33] Lemke A.A., Esplin E.D., Goldenberg A.J., Gonzaga-Jauregui C., Hanchard N.A., Harris-Wai J., Ideozu J.E., Isasi R., Landstrom A.P., Prince A.E.R. (2022). Addressing underrepresentation in genomics research through community engagement. Am. J. Hum. Genet..

[34] H3Africa Community Engagement Working Group (2017). H3Africa Guidelines for Community Engagement (Version Two). https://h3africa.org/wp-content/uploads/2018/05/CE%20Revised%20Guidelines_Final_September%202017%20(1).pdf.

[35] Tindana P., Campbell M., Marshall P., Littler K., Vincent R., Seeley J., De Vries J., Kamuya D., H3Africa Community Engagement Working Group (2017). Developing the science and methods of community engagement for genomic research and biobanking in Africa. Glob. Health Epidemiol..

[36] Tindana P., De Vries J., Campbell M., Littler K., Seeley J., Marshall P., Troyer J., Ogundipe M., Alibu V.P., Yakubu A. (2015). Community engagement strategies for genomic studies in Africa: a review of the literature. BMC Med. Ethics.

[37] Kowal E., Weyrich L.S., Argüelles J.M., Bader A.C., Colwell C., Cortez A.D., Davis J.L., Figueiro G., Fox K., Malhi R.S. (2023). Community partnerships are fundamental to ethical ancient DNA research. HGG Adv..

[38] Lane P., Ferris N., Harrison R., Wilcox M.V. (2014). Rethinking Colonial Pasts through Archaeology.

[39] Lane P.J., Hillerdal C., Karlström A., Öjala C.-G. (2016). Archaeologies of “Us” and “Them”: The Ethics and Politics of Indigeneity in Archaeology and Heritage.

[40] Douglass K., Morales E.Q., Manahira G., Fenomanana F., Samba R., Lahiniriko F., Chrisostome Z.M., Vavisoa V., Soafiavy P., Justome R. (2019). Toward a just and inclusive environmental archaeology of southwest Madagascar. J. Soc. Archaeol..

[41] Bell M., Blue L. (2022). Analysing the Contributions and Longevity of Community Archaeology in the Context of Maritime Cultural Heritage Projects. J. Marit. Archaeol..

[42] Adame F. (2021). Meaningful collaborations can end ‘helicopter research. Nature.

[43] Dalal V., Pasupuleti N., Chaubey G., Rai N., Shinde V. (2023). Advancements and Challenges in Ancient DNA Research: Bridging the Global North–South Divide. Genes.

[44] Haelewaters D., Hofmann T.A., Romero-Olivares A.L. (2021). Ten simple rules for Global North researchers to stop perpetuating helicopter research in the Global South. PLoS Comput. Biol..

[45] Martin A.R., Stroud R.E., Abebe T., Akena D., Alemayehu M., Atwoli L., Chapman S.B., Flowers K., Gelaye B., Gichuru S. (2022). Increasing diversity in genomics requires investment in equitable partnerships and capacity building. Nat. Genet..

[46] Booth T.J. (2019). A stranger in a strange land: a perspective on archaeological responses to the palaeogenetic revolution from an archaeologist working amongst palaeogeneticists. World Archaeol..

[47] Mutsvangwa A., Zezekwa N. (2021). STEM Education: A Ray of Hope for African Countries. Unnes Sci. Edu. J..

[48] Tikly L., Joubert M., Barrett A.M., Bainton D., Cameron L., Doyle H. (2018). Supporting Secondary School STEM Education for Sustainable Development in Africa. https://www.bristol.ac.uk/media-library/sites/education/documents/Supporting%20Secondary%20School%20STEM%20Education%20for%20Sustainabale%20Development%20in%20Africa.pdf.

[49] Hayes V.M., Gong T., Mutambirwa S.B.A., Jaratlerdsiri W., Bornman M.S.R. (2023). African inclusion in prostate cancer genomic studies provides the first glimpses into addressing health disparities through tailored clinical care. Clin. Transl. Med..

[50] Osafo C., Raji Y.R., Burke D., Tayo B.O., Tiffin N., Moxey-Mims M.M., Rasooly R.S., Kimmel P.L., Ojo A., Adu D. (2015). Human Heredity and Health (H3) in Africa Kidney Disease Research Network: A Focus on Methods in Sub-Saharan Africa. Clin. J. Am. Soc. Nephrol..

[51] Zhang C., Hansen M.E.B., Tishkoff S.A. (2022). Advances in integrative African genomics. Trends Genet..

[52] African Union Commission (2015). Agenda 2063: the Africa We Want. https://au.int/sites/default/files/documents/36204-doc-agenda2063_popular_version_en.pdf.

[53] Science benefits from diversity (2018). Nature.

[54] Abungu L. (2005). Museums and communities in Africa: facing the new challenges. Publ. Archaeol..

[55] Gokcumen O., Frachetti M. (2020). The Impact of Ancient Genome Studies in Archaeology. Annu. Rev. Anthropol..

[56] Pollard A.M., Bray P. (2007). A Bicycle Made for Two? The Integration of Scientific Techniques into Archaeological Interpretation. Annu. Rev. Anthropol..

[57] Cunningham J.J., MacEachern S. (2016). Ethnoarchaeology as slow science. World Archaeol..

